# Current knowledge on exosome biogenesis and release

**DOI:** 10.1007/s00018-017-2595-9

**Published:** 2017-07-21

**Authors:** Nina Pettersen Hessvik, Alicia Llorente

**Affiliations:** 1 Department of Molecular Cell Biology, Institute for Cancer Research, Oslo University Hospital, The Norwegian Radium Hospital, 0379 Oslo, Norway; 2 Centre for Cancer Biomedicine, University of Oslo, 0379 Oslo, Norway

**Keywords:** Autophagy, Endosomes, Exosomes, Extracellular vesicles, Lysosomes, Microvesicles, MVB biogenesis, MVBs, Release, Secretion

## Abstract

Exosomes are nanosized membrane vesicles released by fusion of an organelle of the endocytic pathway, the multivesicular body, with the plasma membrane. This process was discovered more than 30 years ago, and during these years, exosomes have gone from being considered as cellular waste disposal to mediate a novel mechanism of cell-to-cell communication. The exponential interest in exosomes experienced during recent years is due to their important roles in health and disease and to their potential clinical application in therapy and diagnosis. However, important aspects of the biology of exosomes remain unknown. To explore the use of exosomes in the clinic, it is essential that the basic molecular mechanisms behind the transport and function of these vesicles are better understood. We have here summarized what is presently known about how exosomes are formed and released by cells. Moreover, other cellular processes related to exosome biogenesis and release, such as autophagy and lysosomal exocytosis are presented. Finally, methodological aspects related to exosome release studies are discussed.

## Introduction

**Fig. 1:**
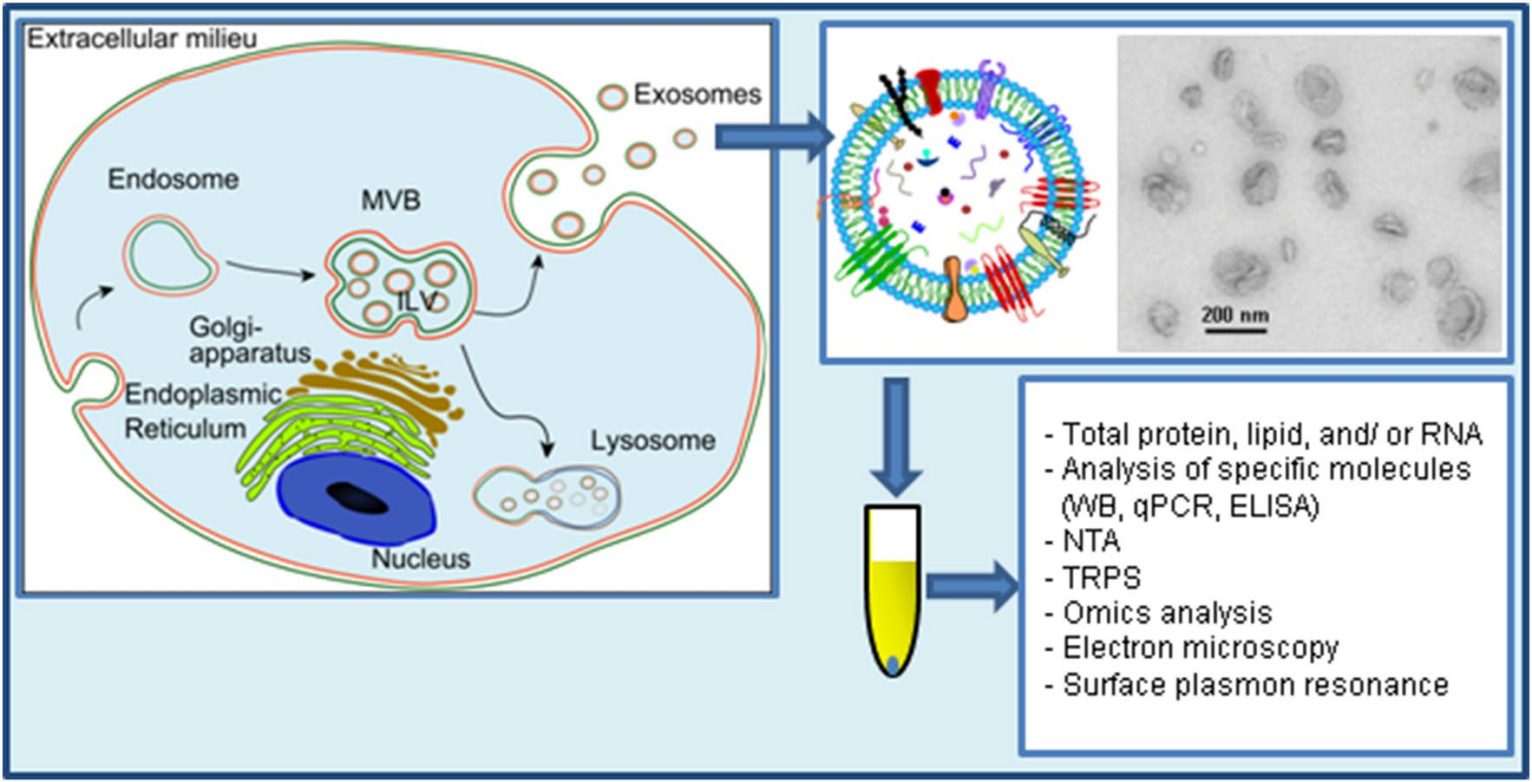
Study of exosome release. Exosomes are released after fusion of MVBs with the plasma membrane. Exosomes have a complex composition of protein, nucleic acids, lipids and other metabolites. Due to their small size (<150 nm in diameter), exosomes are best visualized by electron microscopy. Though some commonly used electron microscopy methods artificially show exosomes as cup-shaped structures, exosomes are round structures. Exosomes are isolated from cell-conditioned media by several protocols that aim to concentrate the exosomal signal to be analyzed and to avoid contaminating molecules such as proteins that are secreted by other mechanisms. Once isolated, exosomes can be analyzed by several methods such as the ones included in this figure. *TRPS* tunable resistive pulse sensing, *NTA* nanoparticle tracking analysis. The figure contains parts reprinted from the Ph.D. degree of Santosh Phuyal, University of Oslo, with permission from the author

Extracellular vesicles (EVs) were first observed 50 years ago in plasma by Wolf, who referred to them as “platelet dust” [1]. Since then, all biological fluids tested have been shown to contain vesicles, and also in vitro grown cell lines have been shown to release vesicles to different extents [2, 3]. These vesicles have received different names during the years, but today are often collectively referred as EVs. Three main types of EVs have been described based on their mechanism of release and size: exosomes (less than 150 nm in diameter), microvesicles/shedding particles and apoptotic bodies (both considered to be larger than 100 nm). The last two types of vesicles are released directly from the plasma membrane in living and dying cells, respectively, and will not be further discussed here. This review deals with the smallest of the family, the exosomes, which are vesicles that are released to the extracellular environment after fusion of late endosomes/multivesicular bodies (MVBs) with the plasma membrane (Fig. 1). This process was first visualized in rat reticulocytes in 1983 [4], and then in sheep reticulocytes in 1985 [5]. Rose Johnstone, a pioneer in the field, chose the term “exosome” in 1987 because “the process seemed to be akin to reverse endocytosis, with internal vesicular contents released in contrast to external molecules internalized in membrane-bound structures” [6, 7]. Further insight into this process has, however, mainly been acquired in recent years [8].

Initially, exosomes were proposed to represent cellular waste [7], and recent data also support the idea of exosomes as an alternative way of eliminating waste products to maintain cellular homeostasis [9, 10]. In addition, these vesicles are suggested to play a role in intercellular communication and have been associated with numerous physiological and pathological functions [2, 11, 12]. Interestingly, exosomes from cancer cells have been shown to promote angiogenesis, modulate the immune system and remodel the surrounding parenchymal tissue, all factors supporting tumor progression (reviewed in [13]). In particular, exosomes have been shown to participate in the generation of the pre-metastatic niche [14–16].

**Fig. 2:**
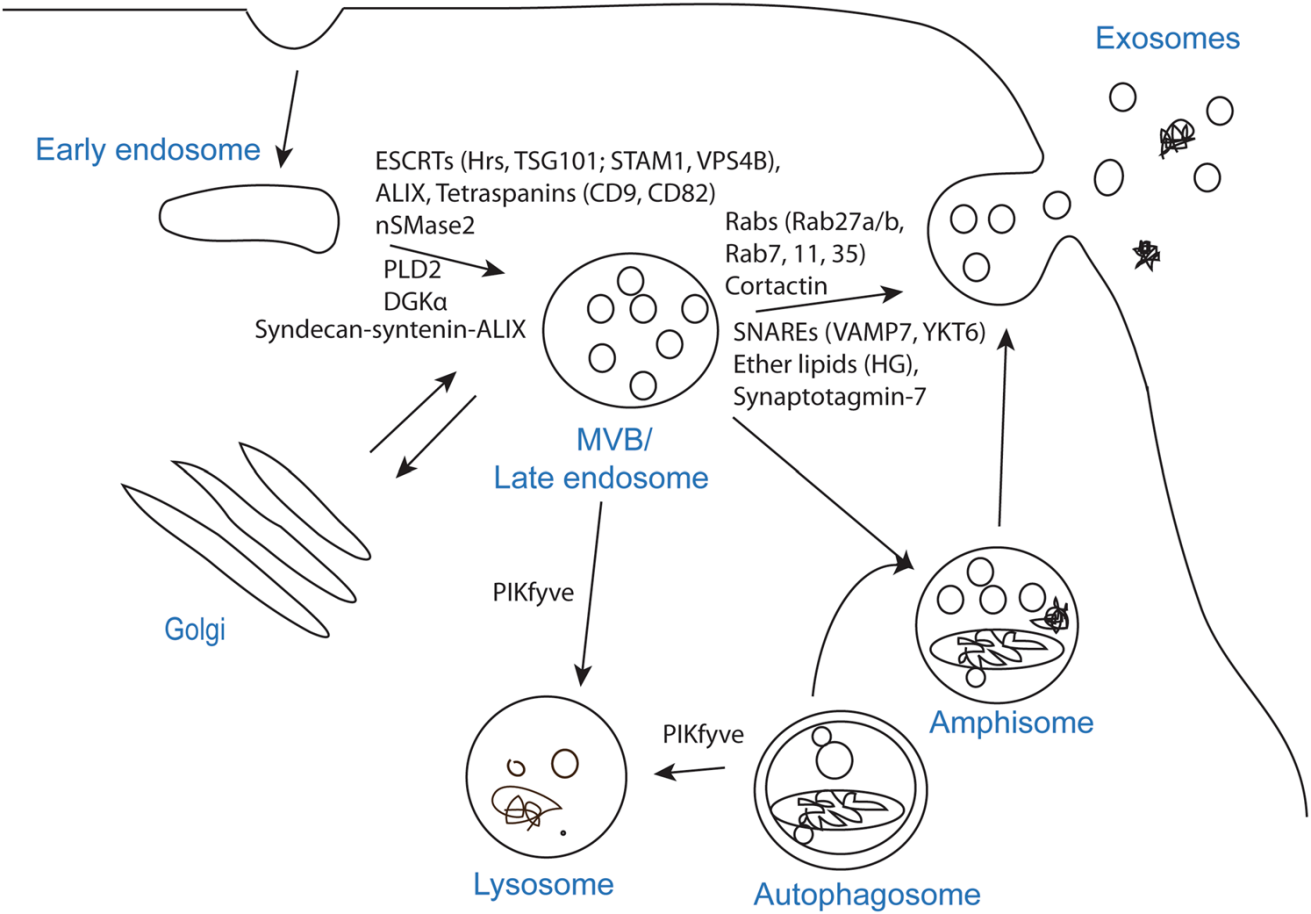
Molecules shown to affect exosome biogenesis and/or release. The process that leads to secretion of exosomes can be divided in three steps; exosome biogenesis, transport of MVBs to the plasma membrane and fusion of MVBs with the plasma membrane. The step affected, or likely to be affected, by each molecule is indicated on the figure

To release exosomes, several cellular steps need to be completed; formation of intraluminal vesicles (ILVs) in MVBs, transport of MVBs to the plasma membrane and fusion of MVBs with the plasma membrane. Several molecules have been implicated in these processes, but due to methodological challenges, it is not easy to distinguish them experimentally, and in many studies it is not clear at which step the investigated molecule/factor operates (Fig. 2). Another important question is whether all MVBs or only specific populations can fuse with the plasma membrane. In agreement with the latter possibility, it has been shown that in B-lymphocytes two pools of MVBs can be identified based on their cholesterol content, and that only MVBs with high cholesterol levels are able to fuse with the plasma membrane and release exosomes [17]. Moreover, EGF and its receptor have been shown to reach a subpopulation of MVBs that are distinct from morphologically identical vacuoles labeled with BMP (bismonoacyl glycerophosphate), also called LBPA (lysobisphosphatic acid) [18], a late endosomal marker [19]. Interestingly, several studies show that exosomes secreted from the apical and basolateral side of polarized cells differ in composition [20–22], thus also supporting the existence of different MVB populations. Furthermore, it would be interesting to learn more about the kinetics of exosome release, for example how many MVBs per hour fuse with the plasma membrane. Measurements of total exosomal protein levels and western blot (WB) analysis of specific proteins indicate that cells only release a small percentage of their content via exosomes. However, as discussed later, the extent of exosome release is cell-dependent, and it can be regulated by different cellular conditions or external factors.

Mass spectrometry-based proteomics and lipidomics analyses have been useful to characterize the proteome and lipidome of exosomes, respectively [23–25]. It can be expected that the composition of exosomes reflects to some extent the composition of MVBs. In fact, proteins associated with MVBs such as several endosomal sorting complex required for transport (ESCRT) proteins or CD63 have been found in exosomes, as can been seen in databases that compile information about the molecular composition of exosomes [26, 27]. Knowledge on the composition of exosomes can give us clues about the machinery involved in their release. However, due to the complex composition of exosomes, it is difficult to identify these molecules. In addition, molecules involved in the release of exosomes do not necessarily need to be incorporated into them.

In this review, the process that ends with the release of exosomes has been divided into several steps (see above) for simplicity. However, it should be mentioned that in some cases the roles of a molecule in a specific step is not completely understood. In addition, a specific molecule can be involved in more than one step along the pathway that leads to the secretion of exosomes. This review also includes a section about methodological issues related to exosome release studies, since they might explain discrepant findings. It is also important to mention that the term “exosomes” is often used to refer to the pellet obtained after 100,000×*g* ultracentrifugation. Although this pellet is probably enriched in exosomes, it might also contain small microvesicles as well as protein aggregates. In fact, it was recently suggested that this pellet should be referred to as small EVs (sEVs), since it was shown to contain different vesicle populations [28]. Therefore, one should be aware that changes in this pellet may not only reflect changes in exosome release.

## Exosome biogenesis

Exosome biogenesis starts within the endosomal system; early endosomes mature into late endosomes or MVBs, and during this process the endosomal membrane invaginates to generate ILVs in the lumen of the organelles [29]. The ESCRT machinery is important in this process. ESCRT consist of four different protein complexes; ESCRT-0, -I, -II, -III and the associated AAA ATPase Vps4 complex [30]. The most thorough study of ESCRTs in exosome biogenesis was an interesting RNAi screen targeting 23 ESCRT and ESCRT-associated proteins in HeLa cells [31]. After shRNA transfection, secreted exosomes were trapped on anti-CD63-beads and detected by anti-CD81 and anti-HLA-DR (MHC II) antibodies using flow cytometry (FC). In this screening, seven ESCRT proteins that affected exosome secretion were identified. Depletion of the ESCRT-0 proteins Hrs and TSG101, and the ESCRT-I protein STAM1 reduced the secretion of exosomes (Table 1; Fig. 2). Knockdown of the ESCRT-III and associated proteins CHMP4C, VPS4B, VTA1 and ALIX increased exosome secretion. When the role of four proteins was verified in exosomes isolated by ultracentrifugation and analyzed by WB, it was found that Hrs, TSG101 and STAM1 depletion decreased exosome secretion, whereas VPS4B knockdown increased it [31]. Silencing of ALIX seemed to change the protein composition of exosomes rather than to affect their secretion. This could indicate that ALIX affects cargo loading and/or the subtypes of MVBs that are destined for secretion. However, the authors showed that ALIX depletion in dendritic cells (DCs) decreased exosome secretion in half of the donors.

**Table 1:** Molecules shown to be involved in exosome biogenesis and/or release

Protein/lipid/modification	Cell line	Secretion quantified by	References
Hrs	DCs, HEK293, SCC61, SCC25-H1047R HNSCC, HeLa	Ubiquitinated proteins, TSG101, VPS4B, Evi, Wnt3A (WB), NTA, CD81/HLA-DR (FC), MHC II, HSC70, CD63 (WB)	[31–34]
STAM1	HeLa	CD81/HLA-DR (FC), MHC II, HSC70, CD63 (WB)	[31]
TSG101	HeLa	CD81/HLA-DR (FC), MHC II, HSC70, CD63 (WB)	[31]
CHMP4C	HeLa	CD81/HLA-DR (FC)	[31]
ALIX	HeLa, DCs, MCF-7	CD81/HLA-DR (FC), CD63, HSP70, syndecan (WB), NTA	[31, 35]
VTA1	HeLa	CD81/HLA-DR (FC)	[31]
VPS4	HeLa	CD81/HLA-DR (FC), MHC II, HSC70, CD63 (WB)	[31]
Syntenin	MCF-7	CD63, HSP70, syndecan (WB), NTA	[35]
Syndecan	MCF-7	CD63, HSP70, ALIX, syntenin (WB), NTA	[35]
CD9	HEK293, BMDCs	β-Catenin, flotillin-1 (WB)	[40]
CD82	HEK293	β-Catenin (WB)	[40]
CD63	HEK293	NTA	[42]
LMP1	HEK293	NTA, acetylcholinesterase activity, Alix, HSC70, CD63, and TSG101 (WB)	[43]
Tspan8	Adenocarcinoma cells	mRNA microarray, RT-qPCR, proteomics, WB	[41]
Synaptotagmin-7	SCC61, SCC25-H1047R HNSCC	NTA	[34]
VAMP7	K562	Acetylcholinesterase activity	[94]
YKT6	HEK293 and A549	TSG101, WNT3A and VPS26/35 (WB)	[33, 95]
Syntaxin 1A	*Drosophila* S2	Evi (WB)	[82]
PKM2	A549, HeLa	NTA	[96]
SNAP-23	A549	NTA	[96]
RalA and RalB	4T1	EM, ALIX, CD63, HSC70 and TSG101 (WB)	[97]
Rab2b	HeLa	HLA-DR (FACS-assay)	[83]
Rab5a	HeLa	HLA-DR (FACS-assay)	[83]
Rab9a	HeLa	HLA-DR (FACS-assay)	[83]
Rab7	MCF-7, HUVEC	CD63, syntenin and syndecan (WB), miR-143 (qPCR)	[35, 89]
Rab11	K562, *Drosophila* S2	Transferrin receptor, Lyn, HSC70 and Evi (WB)	[81, 82]
Rab27a	HeLa, 4T1, TS/A, B-16-F-10, SK-Mel-28, SCC61, SCC25-H1047R HNSCC, Du145	HLA-DR (FACS-assay), total protein, HLA-DR, HSC70, TSG101, CD63, ALIX and LAMP2 (WB), NTA, CD9 (ELISA)	[14, 34, 83, 86, 87]
Rab27b	HeLa, HUVEC	HLA-DR (FACS-assay), total protein, HLA-DR, HSC70 and TSG101 (WB), miR-143 (qPCR)	[83, 89]
Rab35	Oli-neu	PLP (WB)	[84, 85]
Citron kinase	HeLa, 293T	HSC70, CD82, Lamp-1 (WB)	[91]
Cortactin	SCC61	NTA, TSG101, CD63 and flotillin-1 (WB)	[79]
ISGylation	Jurkat T and HEK293	CD63, CD81, TSG101 and flotillin (WB), NTA	[77]
SIMPLE	COS	Fluorescence of LactC2-RFP, NTA, CD63, ALIX (WB)	[44]
nSMase2	Oli-neu, PC-3, HEK293, J77	PLP, EGFP-CD63 (WB), miR-16, miR-146a (qPCR), total protein, CD81 (WB)	[47–49]
DGKα	J-HM1-2.2	CD63, Lamp-1, FasL, (WB)	[55]
PLD2	RBL-2H3, MCF-7	Bodipy-ceramide label (FACS), syntenin, ALIX, CD63 (WB)	[52, 53]
ARF6	MCF-7	Syntenin, ALIX, CD63, SDC1CTF (WB)	[53]
Cholesterol	Oli-neu, PC-3	Flotillin-2, ALIX, EGFP-CD63, PLP-myc, caveolin-1, Lamp-1 (WB)	[100, 101]
Ether lipid (hexadecylglycerol)	PC-3	NTA, total protein	[98]
V-ATPase	HeLa	EM, CD63, ALIX, TSG101 (WB)	[105]
Tetherin	HeLa	EM, CD63, ALIX, TSG101 (WB)	[105]
Hypoxia	MCF-7, SKBR3, MDA-MB 231	NTA, CD63 (WB)	[110]
Irradiation	LNCaP, 22Rv1, PBMC	Vybrant DiI (fluorescent staining), B7-H3 (WB), NTA, total protein	[107, 108]
Cisplatin	A549	Total protein	[109]
PIKfyve	PC-3	NTA, total protein, MS-proteomics	[10]
ER stress (tunicamycin)	MEFs	qNano	[111]
Autophagy (starvation)	K562	Acetylcholinesterase activity, HSC70 (WB)	[113]
ATG12-ATG3	MEFs	Total protein, ALIX, TSG101, GAPDH, HSC70 (WB)	[116]
Autophagy (ATG7)	DCs	GAPDH (WB)	[114]
Intracellular calcium (monensin, A23187, ionomycin)	K562, oligodendrocytes	HSC70 (WB), acetylcholinesterase activity, PLP (WB)	[102, 103]

The methods that were used for exosome quantification in each study are listed

The ESCRT-0 protein Hrs has in addition been shown to play a role in exosome secretion in three other independent studies [32–34]. In the first study, Hrs-depleted DCs were shown to secrete less exosomes, measured as exosomal level of ubiquitinated proteins, TSG101 and VPS4B [32]. Later, Hrs depletion in HEK293 cells was shown to reduce exosomal Wnt3A and Evi secretion [33]. In agreement with this, Hoshino et al. showed by Nanoparticle Tracking Analysis (NTA) that knockdown of Hrs decreased exosome secretion from head and neck squamous cell carcinoma cells [34].

It has been shown that the sorting of syndecans, membrane proteins carrying heparan sulfate chains, in syntenin–ALIX exosomes is mediated by their binding to syntenin. Syntenin is a multivalent soluble protein that also binds ALIX, thus establishing a link between syndecans and the ESCRT machinery [35]. Interesting, this study revealed that in addition to sorting, the interaction between syntenin and ALIX also facilitates ILV formation [35]. The same group later showed that heparanases trim the heparan sulfate side chains of syndecans thus facilitating the formation of syndecan clusters that might stimulate the binding to syntenin [36]. Interestingly, heparanase stimulates the sorting of CD63 too, indicating that the sorting of these two molecules might be related [36]. It should be mentioned that the syndecan–syntenin–ALIX mechanism was estimated to control around 50% of the secreted vesicles in MCF-7 cells [37], in agreement with the idea that different sorting mechanisms may operate in the sorting of exosomal molecules.

Some studies suggest that MVB biogenesis can occur without ESCRTs. For example, it has been shown that despite simultaneously silencing of key subunits of all four ESCRT-complexes, ILVs are still formed in MVBs, thus indicating the presence of ESCRT-independent mechanisms [38]. Tetraspanins, transmembrane proteins enriched in exosomes, are also involved in ESCRT-independent exosome release [39]. Expression of the tetraspanins CD9 and CD82 has been shown to enhance the exosomal release of β-catenin from HEK293 cells [40]. In the same study, the authors also showed that bone marrow dendritic cells (BMDCs) from CD9 knockout mice secrete less exosome-associated flotillin-1. Another tetraspanin that has been shown to be involved in exosome biogenesis is Tspan8 [41]. Expression of Tspan8 in rat adenocarcinoma cells did not affect the total amount of secreted exosomes, but rather changed the mRNA and protein composition of exosomes. Recently, the tetraspanin CD63 was shown to play a role in exosome biogenesis as well [42]. CRISPR/Cas9 knockout of CD63 resulted in reduced secretion of EVs, measured by NTA [42]. The same authors have also shown that cells expressing the Epstein-Barr virus encoded latent membrane protein 1 (LMP1) secreted more exosomes compared to cells not expressing this protein, and that the LMP1-induced particle secretion and packaging into exosomes required CD63 [43].

Another protein that has been suggested to play a role in exosome formation is the small integral membrane protein of the lysosome/late endosome (SIMPLE, also called lipopolysaccharide induced TNF factor, LITAF). Increased secretion of exosomes was observed after transfection of COS cells with SIMPLE, and mutation of SIMPLE interfered with proper MVB formation [44].

In addition to proteins, lipids are also essential players in vesicular transport [45], and both types of molecules collaborate closely in essential processes intrinsic to vesicular transport such as membrane deformation, fission and fusion [46]. Membrane curvature is strongly dependent on the shape of the individual membrane lipids, which depends on the size of the headgroup and on the length and saturation of the acyl chains. Several studies have shown the involvement of lipids in exosome formation by targeting specific lipid modifying enzymes. Inhibition of neutral sphingomyelinase 2 (nSMase2), an enzyme that generates ceramide from sphingomyelin, has been shown to reduce exosomal release of proteolipid protein (PLP) from Oli-neu cells [47]. The same study also showed that inhibition of nSMase2 reduced the release of exosomal EGFP-CD63 from EGFP-CD63-transfected PC-3 cells. The mechanism of this effect is not clear, but it may be due to the formation of ceramide microdomains that coalescence into larger domains that promote membrane budding [47]. It should be mentioned that the role of ceramide in exosome release is not a general one, because it has been reproduced in several [48, 49], but not all [50, 51] cell lines where it has been tested. In PC-3 cells, for example, exosomal release was neither affected by nSMase2 inhibition nor inhibition of de novo synthesis of ceramide [50]. Other lipid modifying enzyme that has been studied in the context of exosome generation is phospholipase D2 (PLD2), an enzyme that produces phosphatidic acid (PA) from phospholipids. The activity of this enzyme was first associated with the release of exosomes in RBL-2H3 cells [52]. A few years later, PLD2 was shown to act as an effector of the small GTPase ADP ribosylation factor 6 (ARF6), which was identified as a regulator of ILV formation and exosome biogenesis [53]. PA, which similarly to ceramide has a small headgroup, may favor membrane invagination by inducing a negative membrane curvature [54], but the direct involvement of PA in the effect PLD2 on exosome biogenesis has not been demonstrated. Finally, diacylglycerol kinase α (DGKα), an enzyme that adds a phosphate group to the lipid second messenger DAG and generates PA, has been shown to regulate the release of exosomes from T lymphocytes [55]. The activity of this kinase seems to play a negative role in the formation of mature MVBs, but also to affect the polarized traffic of MVBs [56]. Moreover, it has recently been shown that the effect of DGKα in the maturation of MVBs and exosome secretion is mediated by protein kinase D1/2 [57].

The biogenesis of exosomes has often been described as an ESCRT-dependent or ESCRT-independent mechanism [2], but the pathways might not be entirely separated [58]. The pathways might work synergistically, and different subpopulations of exosomes could depend on different machineries. Moreover, the cell type and/or cellular homeostasis could be an important factor in the machinery that controls the secretion of exosomes.

## Sorting of cargo into exosomes

Exosomes contain different proteins, lipids and nucleic acids. Their composition is to some extent cell type dependent and can also be influenced by different cellular conditions or treatments. Moreover, exosomes released by a cell line are probably quite heterogeneous [28]. Several studies have described the protein, lipid and RNA cargo of exosomes, but less is known about whether and how the cargo is sorted into the vesicles. Certain miRNAs are enriched in exosomes relative to cells, indicating that miRNAs can be sorted into exosomes [49, 59–61]. Interestingly, a sequence motif that controls the loading of miRNAs through binding to the protein heterogeneous nuclear ribonucleoprotein A2B1 (hnRNPA2B1) has been identified [62]. Exosomal hnRNPA2B1 is sumoylated, and this modification seems to be essential for its miRNA binding [62]. Moreover, KRAS has been shown to play a role in miRNA sorting into exosomes. Exosomes from mutant KRAS colorectal cancer cells show a distinct miRNA profile compared to wild type cells [63]. Furthermore, inhibition of nSMase caused cellular accumulation of certain miRNAs in KRAS mutant, but not wild type cells. Another study has shown that overactive mutated KRAS inhibits localization of the RISC component Argonaute 2 (Ago2) to MVBs and decreases Ago2 secretion in exosomes [64]. Inhibition of mitogen-activated protein kinase kinases (MEKs) I and II was shown to reverse the effect of the activating KRAS mutation, leading to increased exosomal Ago2 secretion. mRNAs also seem to be selectively enriched in exosomes [65]. Exosomal mRNAs show enrichment in 3′UTR fragments [66], which could play a role for mRNA sorting into the vesicles [67]. Exosomes have also been shown to contain ubiquitinated proteins [10, 68], and ubiquitination could be a mechanism to target proteins to exosomes [69, 70].

Lipids may also be important for sorting of specific proteins into exosomes. Interestingly, exosomes have been shown to be enriched in cholesterol, sphingomyelin and glycosphingolipids compared to their parent cells [71, 72]. This suggests that exosomal membranes may contain lipid rafts, membrane subdomains enriched in cholesterol and glycosphingolipids that play important roles in signaling and sorting [73, 74]. In fact, one of the first studies on the role of lipids in exosome release showed that lyn, flotillin-1 and stomatin are released to the extracellular medium via their association with lipid domains (Triton X-100-insoluble fractions) in the exosomal membrane [75]. In addition, sphingosine 1-phosphate (SP1), a lipid formed by the phosphorylation of sphingosine by sphingosine kinase 1 (Sphk1) and 2 (Sphk2), has been shown to regulate cargo (such as CD63, CD81 and flotillin) sorting into exosomes via inhibitory G protein (Gi)-coupled S1P receptors located on MVB membranes [76]. These receptors are continuously activated through a constant supply of S1P catalysed by sphingosine kinase (SphK), though it is not clear how the kinase is recruited to MVBs. Importantly, impairment of S1P signalling did not reduce the total number or size of exosomes, even if secreted exosomes contained lower amounts of CD63. This suggests that S1P signalling is mainly involved in sorting of cargo molecules into exosomes, and not in ILV formation [76].

## Transport of MVBs to the plasma membrane and exosome release

Multivesicular bodies can either be directed to lysosomes where their content is degraded or transported to the plasma membrane for exosome release. Little is known about the molecular mechanisms and the cellular statues that regulate this balance. Recently, ISGylation, a posttranslational ubiquitin-like modification, was proposed to be one of the signals regulating the MVBs’ fate [77]. Induction of ISGylation was shown to impair exosome secretion, measured by quantification of several exosomal markers by WB, as well as by NTA. The authors suggested that ISGylation of MVB proteins promotes fusion of MVBs with lysosomes, thereby directing MVBs to the degradation pathway and away from the secretory pathway [77].

Transport of MVBs to the plasma membrane is dependent on their interaction with actin and the microtubule cytoskeleton [34, 49, 78]. Knockdown or overexpression of the actin binding protein cortactin has been shown to decrease or increase exosome release, respectively [79]. Moreover, live-cell imaging indicated that cortactin is involved in both trafficking and docking of MVBs to the plasma membrane [79]. Rab GTPases, the largest family of small GTPases [80], regulate many steps of membrane trafficking, including vesicle budding, transport of vesicles along actin and tubulin, as well as membrane fusion. Interestingly, several Rab GTPases have been shown to play a role in exosome secretion, although their precise mechanism of action in this process is not known. The first Rab GTPase shown to be involved in exosome secretion was Rab11. Overexpression of a dominant-negative Rab11 mutant inhibited exosome release, measured by quantification of the exosomal levels of transferrin receptor, Lyn and HSC70, in human leukemic K562 cells [81]. In line with this, depletion of Rab11 in *Drosophila* S2 cells reduced the release of Evi-bearing exosomes [82]. On the contrary, Rab11 was not found to affect exosome release from HeLa cells [83]. Hsu et al. showed that knockdown of Rab35 decreased the release of exosome-associated proteolipid protein (PLP) from the oligodendroglial cell line Oli-neu, possibly due to reduced docking/tethering of MVBs at the plasma membrane [84]. Reduced exosome release after Rab35 knockdown was later confirmed by Fruhbeis et al. using the same model [85], whereas Rab35 depletion did not affect exosome release from *Drosophila* S2 cells [82].

In a shRNA screening targeting 59 Rab GTPases in HeLa cells, five of them were found to be involved in exosome secretion [83]. After shRNA transfection, secreted exosomes were trapped on anti-CD63-beads and detected by anti-CD81 and anti-HLA-DR (MHC II) antibodies using FC. Knockdown of Rab2b, Rab5a, Rab9a, Rab27a and Rab27b decreased exosome secretion in the screening assay. The effect of Rab27a and Rab27b was verified by measuring the total amount of exosomal protein, as well as the exosomal level of HLA-DR, HSC70 and TSG101. Ostrowski et al. also showed by total internal reflection fluorescence (TIRF) microscopy that Rab27a and Rab27b depletion reduced docking of MVBs to the plasma membrane [83]. The effect of Rab27a on exosome release has been reported in several studies using different cell lines [14, 34, 86, 87]. Nevertheless, some studies do not show an effect of Rab27 knockdown on exosome release [82, 88]. The last Rab protein that has been shown to be involved in exosome secretion is Rab7. Baietti et al. showed that Rab7 regulates secretion of syntenin and syndecan-containing exosomes from MCF-7 cells, whereas no effect was observed in HeLa cells [35, 83]. Moreover, Rab7 and Rab27b, but not Rab27a, have been shown to regulate secretion of exosomal miR-143 from HUVEC cells [89]. Contrary to previous studies, a recent report showed that knockdown of Rab27b increased particle release from PC-3 cells [90]. However, in this study exosomes were not isolated and the conditioned media was measured by NTA directly after 12,000×*g* centrifugation. The discrepant findings might be due to cell-specific regulations, or due to methodological challenges, such as different protocols for isolation of vesicles and different methods to quantify the amount of released exosomes (discussed below).

Small GTPases of other families such as the Rho/Rac/cdc42 family might also play a role in exosome release. In particular, in a study of RhoGTPases in HIV-1 virion production, the RhoA effector citron kinase was shown to increase the release of exosomes [91].

Exosomes are released into the extracellular environment upon fusion of MVBs with the plasma membrane. During this process several energy barriers need to be overcome. A number of protein–lipid and protein–protein interactions have been shown to reduce these energy barriers and facilitate fusion, as well as to provide specificity. Proteins involved in membrane fusion include soluble *N*-ethylmaleimide-sensitive factor attachment protein receptors (SNAREs), tethering factors, Rabs, and other Ras GTPases [92]. The specific molecular machinery for fusion of MVBs with the plasma membrane is not well characterized.

SNARE proteins facilitate fusion of vesicles with their target membrane, such as the plasma membrane or the membrane of different organelles [93]. A SNARE complex is built up by three or four SNARE proteins forming four coiled-coil helices. The members of this protein family are classified as either R- or Q-SNAREs. Generally, fusion involves one R-SNARE (usually v-SNARE) and three Q-SNAREs (usually t-SNAREs) [92]. Fader et al. showed that the R-SNARE vesicle-associated membrane protein 7 (VAMP7) is necessary for exosome release in the human leukemic cell line K562 [94]. In this paper, overexpression of the N-terminal domain of VAMP7, which inhibits SNARE complex formation, reduced exosome release, measured as exosome-associated acetylcholinesterase. The authors also observed that the MVBs were enlarged and distributed to the cell periphery after overexpression of the N-terminal domain of VAMP7, thus suggesting that the fusion of MVBs with the plasma membrane was impaired [94].

Another R-SNARE protein, YKT6, has been shown to be required for exosome release in two independent studies. Gross et al. showed that depletion of YKT6 decreased the level of TSG101, WNT3A and VPS26/35 in exosomes secreted from human embryonic kidney HEK293 cells [33]. In line with this, Ruiz-Martinez et al. showed a reduced level of exosome-associated TSG101 after knockdown of YKT6 in A549 human lung cancer cells [95]. In *Drosophila* S2 cells, depletion of the Q-SNARE syntaxin 1A (Syx1A) decreased release of Evi-bearing exosomes [82]. Recently, pyruvate kinase type M2 (PKM2) was shown to phosphorylate SNAP-23, which in turn enables exosome release [96].

A study in *Caenorhabditis elegans* indicated that the Ras-related GTPase homolog (Ral-1) is involved in MVB formation and fusion with the plasma membrane [97]. Likewise, in 4T1 mouse mammary tumor cells, knockdown of the mammalian homologs Ras like proto-oncogene A (RalA) and RalB reduced the secretion of exosome-like vesicles. In that study exosome release was quantified by electron microscopy (EM) and by the exosomal level of ALIX, CD63, HSC70 and TSG101 [97]. Interestingly, the authors also showed that MVBs accumulate under the plasma membrane when the Q-SNARE syntaxin 5 was absent in *C. elegans.*

Furthermore, addition of an ether lipid precursor that increases the levels of cellular (and exosomal) ether lipids has been shown to increase the release of exosomes from PC-3 cells [98]. The mechanism of this effect is not clear, but ether lipids have previously been suggested to be involved in membrane fusion [99], and the increased exosome secretion could be due to facilitated fusion of MVBs with the plasma membrane. Moreover, addition of cholesterol (in complex with methyl-beta cyclodextrin) in Oli-neu cells has been shown to increase the exosomal levels of several proteins such as flotillin-2, ALIX and CD63 [100]. In contrast, a reduction of cholesterol levels in PC-3 cells, both by addition of methyl-beta cyclodextrin and by metabolic inhibition of its formation, increased the secretion of several exosomal proteins [101].

Importantly, it has been shown that exosome release can be regulated by calcium. Increased intracellular calcium level after treatment of human erythroleukemia K562 cells with monensin or the calcium ionophore A23187 has been shown to increase exosome secretion [102]. In line with this, the calcium ionophore ionomycin was shown to facilitate exosomal release of PLP from oligodendrocytes [103]. Some proteins, such as synaptotagmins, function as calcium sensors and have been implicated in vesicular transport [104]. Interestingly, a member of the synaptotagmin family has been reported to affect exosome release. Knockdown of synaptotagmin-7 was shown to reduce exosome secretion, as measured by NTA [34], likely by affecting the fusion of the MVBs with the plasma membrane.

Interestingly, it has been shown that inactivation of the vacuolar ATPase (V-ATPase) in HeLa cells results in increased exosome secretion, and that the exosomes remain clustered and attached to the plasma membrane by tetherin [105]. This indicates that these exosomes may not reach long distances and may stay closely attached to the secreting cell. Regulation of tetherin expression could be a cellular mechanism to regulate whether exosomes should exert their effect locally or at longer distances.

## Cellular homeostasis affects exosome release

It has been suggested that the destination of MVBs to either degradation or secretion depends on cellular homeostasis and that exosomes play a role in protecting cells against intracellular stress [9, 106]. Several studies have shown that cellular stress increases exosome secretion [107–111]. Irradiation of cells has been shown to induce senescence and to increase exosome release [107, 108]. Increased exosome release has also been reported after cisplatin treatment [109], as well as after exposure to hypoxia [110]. Furthermore, induction of ER stress by tunicamycin increased the number of cellular MVBs and enhanced exosome secretion [111]. It is not clear why cells respond to stress by releasing more exosomes, but this could be an alternative way of eliminating waste products. The secreted exosomes might be targeted to and degraded by phagocytes, but they might also have other destinations. Exosomes secreted as waste are likely to affect neighboring cells and possibly induce pathological conditions. Another possibility is that cells might communicate to neighboring cells about intracellular stress by increasing exosome release.

Similarly to the stress hypothesis, a link between autophagy and exosomes has been proposed [9]. Autophagy is a degradative pathway that supplies nutrients during starvation and eliminates damaged organelles, aggregated proteins and invading pathogens [112]. This pathway can be induced by various stimuli to maintain cellular homeostasis. Upon autophagy induction cytoplasmic cargo is trapped within double-membrane vesicles termed autophagosomes, which can fuse with MVBs to form amphisomes or directly with lysosomes [112]. The cargo is then degraded in the lysosomes and the components are transported back to the cytoplasm. Fader et al. showed that induction of autophagy by starvation reduced exosome release [113]. The authors proposed that this was caused by increased fusion of MVBs with autophagosomes, thereby directing MVBs to the degradative pathway. Moreover, inhibition of autophagy by ATG7 depletion has been shown to enhance exosomal secretion of GAPDH [114]. Recently, we showed that inhibition of the phosphoinositide kinase PIKfyve, which generates phosphatidylinositol-3,5-bisphosphate, increased exosome release and reduced autophagic degradation, most likely due to a reduced fusion of lysosomes with both MVBs and autophagosomes [10]. Rather than an effect of autophagy *per se* on exosome release, this could be due to interference with transport or fusion of the organelles. In line with this, it has also been shown that lysosomal dysfunction induced by ammonium chloride or bafilomycin A1 leads to increased secretion of alpha-synuclein in exosomes from SH-SY5Y cells [115]. In such cases where the transport through the degradative pathway is obstructed or the lysosomal pathway is overloaded due to stress, exosome release might indeed be an alternative route to dispose waste.

Murrow et al. showed that basal autophagy is impaired in cells lacking ATG12-ATG3, a conjugate of two autophagy-related proteins, whereas starvation-induced autophagy is not affected [116]. The authors also showed that ATG12-ATG3 deficiency impaired exosome release, measured as total exosomal protein as well as by the levels of several exosomal markers, and suggested this was due to impaired late endosomal function. This could be another mechanism linking exosomes to autophagy.

In addition to its more thoroughly studied role in degradation, the autophagic machinery is also involved in a less known process termed secretory autophagy [117]. Secretory autophagy is considered as an unconventional secretion process that releases numerous cytoplasmic substrates from the cell [117]. Since secretory autophagy is induced in cells with lysosomal dysfunction this might be, in a similar way as exosomes, an alternative way of eliminating waste products. Certain neurodegenerative diseases are associated with dysfunctional autophagy and deposition of aggregation-prone proteins, thus secretory autophagy might play a role in these diseases [118, 119].

Furthermore, not only MVBs, amphisomes and autophagosomes can release their content to the extracellular environment, but also lysosomes in a process called lysosomal exocytosis [120]. These secretion pathways indeed share some features and do somewhat overlap. When a cell is no longer able to degrade material in the lysosomes due to a lysosomal defect, lysosomal overload or transport interference, secretion of the content of lysosomes, MVBs or amphisomes could be a way to rescue the cell. Furthermore, when amphisomes fuse with the plasma membrane, the ILVs included in the amphisome will appear extracellularly as exosomes. Also if a lysosome secretes its content before it is fully degraded, remaining ILVs can be released as exosomes. Though these pathways cannot be entirely separated, each pathway is probably regulated by distinct machineries.

## Methodological aspects related to studies of exosome release

There is no consensus in the literature about the optimal methods and conditions to study exosome biogenesis and release. The methodology is continuously being developed as new technological advances appear and new knowledge is generated. In this section, we describe the different parameters that have to be considered and the different methods that are used to study exosome release. Importantly, different methodological approaches might explain reproducibility issues. It should be mentioned that this section is not meant to give an overview of the exosome methodology in general (for reviews on the subject see [121–126]), but to present methodological aspects related to exosome biogenesis and release studies.

### Cellular models

Studies of exosome biogenesis and release have mainly been performed using in vitro grown cell lines as a model. There are few published comparative studies [127], but different cell lines can be expected to release different levels of exosomes to the extracellular environment. It should also be mentioned that some cell lines may have cell-specific molecular machineries for exosome biogenesis and release. Therefore, in order to generalize the function of a specific molecule in exosome release, several cell lines and readouts of exosome release have to be tested.

### Exosome collection

The amount of exosomes needed in each experiment depends on the sensitivity of the quantification method. Based on this and on the cell line that is going to be used, the number of cells per experiment and/or the collection time of exosomes can be adjusted. Kinetic studies of exosome release are not often seen in the literature. Authors often choose a specific collection time that varies from a few hours to several days. We have observed that there is an increasing release of exosomes during the first 24 h in PC-3 cells [101]. The medium in which the exosomes are collected is also an important factor. Many cell lines are grown in the presence of fetal calf serum, which naturally contains exosomes, and thus may affect the results. To avoid this problem, exosomes are either collected in medium without serum, if the cells tolerate this, or with medium depleted of exosomes [122]. It should be mentioned that other medium supplements, such as bovine pituitary extract, even though they might not contain exosomes, might contain other vesicles or proteins that may be pelleted with exosomes during ultracentrifugation.

### Exosome isolation

Exosomes have often been isolated by sequential centrifugation in exosome release studies. This method includes 2–3 centrifugations at low speeds to remove cells, cell debris and microvesicles, followed by ultracentrifugation at approximately 100,000×*g* for 1–2 h and a PBS wash [122]. It cannot be expected that this method gives a pure exosome population because the size of exosomes and microvesicles overlaps to some extent, but a fraction that is enriched in exosomes. Importantly, it has recently been shown that the 100,000×*g* pellet could be divided into several categories both by floatation into iodixanol gradients and by immunoisolation using beads coated with antibodies targeting either CD9, CD63, or CD81 [28]. Based on these results it was suggested that vesicles in the 100,000×*g* pellet should be referred to as “small EVs” (sEVs) instead of exosomes [28]. Moreover, if present, lipoparticles from serum and lipid droplets would be co-isolated together with exosomes during ultracentrifugation [71]. Autophagosomes can contain lipid droplets in their lumen that would be released from the cells and co-isolated with exosomes if secretory autophagy is induced [10]. As recently discussed in another review, the presence of large amounts of triacylglycerol or cholesteryl ester in exosome preparations is an indication of contamination with lipid droplets or lipoproteins [71]. The heterogeneity of the 100,000×*g* pellet and the presence of potential contaminations are likely to have caused some of the discrepancies reported in exosome release studies. Another drawback of ultracentrifugation is that the throughput is limited by the rotor capacity.

Although differential centrifugation is the most common method used for exosome isolation, other methods can be used to obtain exosome or exosome-enriched samples. Density gradient centrifugation was shown to give the purest exosome population when compared to ultracentrifugation and precipitation-based methods [121]. However, this method gives a relatively low yield and is time consuming. Moreover, Kowal et al. reported that density gradient centrifugation of 100,000×*g* pellets was not able to clearly separate subpopulations of small EVs [28]. Immunoisolation of exosomes based on specific proteins at the exosomal membrane is also a method that would result in relatively pure EV subpopulations [28, 128], but it requires that the selected membrane protein is carefully chosen and that the immunoisolation protocol is optimized. Interestingly, Tauro et al. reported that immunoaffinity capture was more efficient to isolate exosomes from a colon cancer cell line than ultracentrifugation and density gradient isolation [129]. Surface plasmon resonance (SPR) also allows analyzing specific exosome populations. SPR-based quantification of exosomes is based on the capture of exosomes on an immuno-functionalized surface and measurements of the resulting change in refractive index. This methodology has shown promising results in detecting exosomes containing specific exosomal markers such as CD63 as well as cancer specific proteins [130–132]. Another isolation technique is based on the precipitation of exosomes with volume-excluding polymers such as polyethylene glycol (PEG). This is a rapid method, but that probably results in the coisolation of exosomes with other structures of similar sizes. Finally, a method that is gaining popularity in the EV field is size exclusion chromatography (SEC). This method allows the separation of exosomes from proteins, but not from microvesicles, protein aggregates and lipoparticles.

Whether it is beneficial to isolate specific exosome subpopulations rather than all small vesicles remains a question for debate. At the present stage, there is little knowledge about the function of the different EV subpopulations. The EV subpopulations, and their protein markers, might also be highly dependent on the cell type, as well as the homeostasis and treatment of the cell. Thus, one should be careful to apply strict guidelines for categorizing EV subpopulations at this point. The optimal isolation method will depend on the aim of the study, the downstream analysis and which impurities are acceptable in that context. Importantly, it seems clear that the choice of isolation method can affect the results obtained from studies on exosome biogenesis and release.

### Exosome analysis

To investigate whether a specific treatment or molecule affects exosome release, the number of vesicles released has to be determined (Fig. 1). In many cases, this has been measured in an indirect manner for example by measuring the enzymatic activity of a specific exosomal protein or the levels of typical exosomal proteins such as TSG101 or ALIX, or specific proteins (PLP) (Table 1). These methods should be supplemented with additional methods and/or additional molecules since alterations in the cellular levels or sorting of the chosen protein by the treatment might affect the results. Moreover, Kowal et al. have shown that the 100,000×*g* pellet contains subpopulations of vesicles with different protein composition [28]. In addition, the authors found that some of the proteins commonly used as exosomal markers are also present on bigger vesicles recovered in the 2000×*g* and the 10,000×*g* pellets. Based on their results, the authors concluded that syntenin-1 and TSG101 can be considered specific markers of bona fide exosomes. It is likely that the analysis of only one or a few proteins, some of which have retrospectively not been shown to be optimal exosomal markers, in studies of exosome release might have affected the conclusions drawn. Mass spectrometry studies appear as a good alternative since they allow a more general characterization of the exosome proteome.

A more general, but still indirect method to analyze exosome release is to measure the total amount of protein (total amount of lipid or RNA can also be measured) in exosomes. However, this requires that the treatment does not change the vesicle size or content. In addition, the method requires that the exosome samples are not contaminated by protein aggregates. Methods that combine measurement of the total protein and lipid content of exosomes have also been tested [133]. FC after capture on beads has been used in several studies [83, 134], and direct FC has also become an option using specific methods or instruments with small particle detection capability [135]. Recently, NTA and tunable resistive pulse sensing (TRPS) have emerged as new methods to measure exosome concentration [136–138] and have been used in exosome release studies [10, 139]. These can be considered as direct methods since they measure the concentration of particles in solution. However, one should be aware that they do not exclusively measure exosomes, and that particles of similar sizes in the samples such as aggregates or virus will be counted. Another technique that allows quantification of exosomes is SPR, as mentioned above. In conclusion, several methods are available to directly or indirectly measure the number of exosomes released, and a combination of several methods is recommended to confirm the implication of a molecule in the process. However, exosome release is the result of several steps, and quantifying the released exosomes does not indicate which step is affected. More insight can be obtained by studying MVB morphology, number, location and ability to fuse with the plasma membrane. A few studies have addressed this issue by using immunofluorescence microscopy [83] or electron microscopy [10, 84, 98], but further methodological developments are required to study the different steps independently.

## Conclusion

Several molecules have been implicated in exosome biogenesis and release, but for many of them, the exact mechanism of action is not clear yet. Exosome biogenesis and secretion seem to utilize several machineries, depending on either cell type or cellular homeostasis. In addition, different MVB subtypes might exploit different pathways. Finding consensus in the field is complicated by methodological challenges such as the use of different methods for exosome isolation and quantification. However, this is an interesting field that needs to be further explored. Moreover, considering the role of exosomes in physiological and pathological conditions, strategies that interfere with the release of exosomes and impair exosome-mediated cell-to-cell communication could potentially be exploited therapeutically in the future.

## Abbreviations

EM: Electron microscopy
ESCRT: Endosomal sorting complex required for transport
EVs: Extracellular vesicles
FC: Flow cytometry
ILV: Intraluminal vesicle
MVB: Multivesicular body
NTA: Nanoparticle tracking analysis
SNAREs: Soluble *N*-ethylmaleimide-sensitive factor attachment protein receptors
WB: Western blot
